# Renal Phosphate Reabsorption is Correlated with the Increase in Lumbar Bone Mineral Density in Patients Receiving Once-Weekly Teriparatide

**DOI:** 10.1007/s00223-015-0073-7

**Published:** 2015-10-19

**Authors:** Yasuhiro Takeuchi, Tatsuhiko Kuroda, Toshitsugu Sugimoto, Masataka Shiraki, Toshitaka Nakamura

**Affiliations:** Toranomon Hospital Endocrine Center, 2-2-2 Toranomon, Minato-Ku, Tokyo 105-8470 Japan; Medical Affairs Department Asahi Kasei Pharma Corporation, 1-105 Kanda Jinbocho, Chiyoda-Ku, Tokyo 101-8101 Japan; Internal Medicine 1, Shimane University Faculty of Medicine, 89-1 Enya-cho, Izumo, Shimane 693-8501 Japan; Research Institute and Practice for Involutional Diseases, 1610-1 Meisei, Misato, Azumino, Nagano 399-8101 Japan; National Center for Global Health and Medicine, 1-21-1 Toyama, Shinjuku-Ku, Tokyo 162-8655 Japan; Okinaka Memorial Institute for Medical Research, 2-2-2 Toranomon, Minato-Ku, Tokyo 105-8470 Japan

**Keywords:** Teriparatide, Phosphate, Reabsorption, Bone mineral density, Osteoporosis

## Abstract

In order to assess the changes in serum calcium and phosphate and the changes in renal tubular phosphate reabsorption (TmP/GFR) and to evaluate the association between these indices and the increase in bone mineral density (BMD) with once-weekly intermittent administration of teriparatide (TPTD), the results from the teriparatide once-weekly efficacy research (TOWER) trial were re-analyzed. The TOWER trial studied postmenopausal women and older men with osteoporosis. Patients were randomly assigned to receive TPTD 56.5 μg or placebo for 72 weeks. Of these patients, the present study investigated those whose calcium and phosphate levels and lumbar BMD (L-BMD) were measured (TPTD group, *n* = 153 and Placebo group, *n* = 137). The TPTD group had significantly lower serum phosphate, calcium-phosphate product, and TmP/GFR at weeks 4, 24, 48, and 72 and urinary fractional calcium excretion (FECa) at weeks 12, 48, and 72 (*p* < 0.05). In the TPTD group, the serum phosphate and TmP/GFR during early treatment (4, and 12 weeks) showed a significant positive correlation with the percent change in L-BMD at weeks 48 and 72. Based on multivariate analysis corrected for age, BMI, and L-BMD at the start of treatment, serum phosphate and TmP/GFR at week 4 showed a significant correlation with the percent change in L-BMD. This study suggests that the L-BMD response to once-weekly long-term TPTD treatment is associated with circulating phosphate or with the status of its renal reabsorption. Preventing decrease in serum phosphate levels may be important in acquiring greater L-BMD with once-weekly TPTD.

## Introduction

The homeostasis of circulating calcium and phosphate plays an important role in bone mass regulation. Calcium homeostasis is primarily regulated by parathyroid hormone (PTH), which mediates calcium reabsorption at the renal distal tubule and calcium mobilization from the bone via bone resorption and also promotes calcium absorption from the intestinal tract by vitamin D mediated through vitamin D activation by PTH at the renal proximal tubule [1]. PTH mobilizes phosphate from the bone through promoting bone resorption and promotes phosphate absorption from the intestinal tract via vitamin D activation [2, 3]. On the other hand, PTH is also known to suppress phosphate reabsorption at the renal proximal tubule and promote the secretion of FGF23, a phosphate diuretic hormone [4]. Collectively, PTH is known to suppress phosphate reabsorption in the kidneys and decrease the circulating phosphate concentration. Since calcium deficiency secondarily induces PTH oversecretion, PTH in turn promotes bone resorption and decreases circulating phosphate concentration, leading to decreased bone mass and bone mineral density (BMD) [5]. Moreover, phosphate deficiency blocks the formation of hydroxyapatite, which is a calcium phosphate crystal found locally in bone, leading to decreased BMD. On the other hand, although the secretions of PTH and FGF23 are stimulated in response to phosphate oversupply [6], excessive phosphate can cause tunica media thickening or vascular calcification when adequate compensation cannot be achieved [7] and is consequently known to be associated with the incidence of cardiovascular events or heart failure [8–10].

Teriparatide (TPTD) is an analog of PTH (1-34) with pharmacological actions similar to PTH. Its intermittent daily or once-weekly administration is known to increase BMD and significantly suppress fractures [11, 12].

When a single dose of TPTD 56.5 μg was administered, serum calcium increased transiently from 9.11 to 9.58 mg/dL around 4 to 6 h after the injection and subsequently returned to its baseline concentration until 24 h after the injection. At the same time, a decrease in urinary calcium excretion was observed at 4 h and again the decrease was transient. After TPTD administration, an increase in urinary phosphate excretion was observed at 2 and 6 h after the drug injection, but the increase was transient. Serum phosphate level was decreased transiently as a result, but subsequently returned to the level that was not statistically different from that of baseline value [13]. When patients receive TPTD 56.5 μg once-weekly repeatedly, serum calcium changes similarly, but without attenuation in its effects for 24 weeks [14]. On the other hand, the serum phosphate level just before TPTD administration gradually decreases compared to the level at the start of treatment. Whether these changes in calcium and phosphate during once-weekly intermittent TPTD treatment are associated with the increase in BMD has not been previously assessed.

Recently, the associations among changes in calcium and phosphate, changes in BMD, and changes in intima-media thickness (IMT) with daily TPTD treatment were investigated. The results showed that the changes in calcium and phosphate were not significantly correlated with BMD [15].

In the present study, to clarify whether the earlier changes in calcium and phosphate metabolism can predict the later change of BMD, the changes in serum calcium and phosphate, the changes in renal tubule phosphate reabsorption, and the association between these changes and the increase in BMD were evaluated in patients who received once-weekly TPTD.

## Materials and Methods

### TOWER Trial

Data from the TOWER trial, a randomized, controlled trial conducted in Japan with the objective of determining the effects of TPTD on suppressing bone fractures in postmenopausal women and men with osteoporosis, were used in the present analysis [12]. Patients were randomly assigned to receive once-weekly subcutaneous injections of TPTD 56.5 μg or placebo for 72 weeks. All patients received daily oral supplements of calcium 610 mg, vitamin D 400 IU, and magnesium 30 mg.

### Clinical Laboratory Measurements

Blood and urine samples were collected prior to each TPTD treatment. Samples were analyzed collectively at a central laboratory. Adjusted serum calcium, serum phosphate, serum creatinine, urinary calcium, urinary phosphate, and urinary creatinine concentrations were measured at weeks 0, 4, 24, 48, and 72. At each time point, adjusted serum calcium-phosphate product, urinary fractional calcium excretion (FECa), and the phosphate reabsorption index TmP/GFR were calculated. Equations for FECa and TmP/GFR are as follows:

FECa (%) = (Urinary calcium × Serum creatinine) × 100/(Urinary creatinine × Serum calcium)

TmP/GFR = Serum phosphate × (1 − (Urinary phosphate × Serum creatinine)/(Serum phosphate × Urinary creatinine))

### BMD Measurement

Lumbar (L)-BMD was measured at each medical institution at weeks 0, 24, 48, and 72, and DXA data of the L2-4 area were determined using two types of instruments [QDR (Hologic, Bedford, MA) and DPX (GE Healthcare, Fairfield, CT)]. DXA data were re-analyzed collectively at a central laboratory and measured by a specialist. Percent changes between late treatment (weeks 48 and 72) and pre-treatment levels were calculated.

### Statistical Analysis

All continuous variables are expressed as mean ± standard deviation (SD). Adjusted serum calcium, serum phosphate, serum calcium-phosphate product, FECa, and TmP/GFR were compared between groups for each time point (*t*-test). The associations between these variables during early treatment (weeks 4 and 12) and the percent change in L-BMD during late treatment (weeks 48 and 72) were evaluated using Pearson’s correlation coefficient. Variables that showed significant correlations with L-BMD were subjected to multivariate analysis using age, BMI, and initial L-BMD as factors for adjustment. Additionally, significant explanatory variables were split at the median of the TPTD group into high and low groups, and the difference in the actual percent changes in L-BMD was compared with the *t*-test between the two groups for each variable. *p* < 0.05 was considered significant.

## Results

Of the 578 patients who received randomized treatment in the TOWER trial, those whose clinical laboratory values of calcium and phosphate, as well as L-BMD, were measured (TPTD group, *n* = 153; Placebo group, *n* = 137) were investigated. The patient characteristics of each group are shown in Table 1. Significant differences were not evident between the groups in any of the parameters.

**Table 1:** Baseline characteristics

Item	Placebo (*n* = 153)		TPTD (*n* = 137)		*p*
	Mean	SD	Mean	SD	
Age (years)	74.9	5.9	74.2	5.4	0.348
BMI (kg/m^2^)	23.0	3.2	22.9	3.1	0.823
Adjusted serum calcium (mg/dL)	9.6	0.4	9.5	0.4	0.237
Serum phosphate (mg/dL)	3.6	0.4	3.6	0.5	0.671
Adjusted serum calcium × phosphate	34.6	4.9	34.2	4.7	0.459
FECa (%)	1.3	0.8	1.3	0.8	0.520
TmP/GFR (mg/dL)	3.3	0.5	3.2	0.4	0.553
L-BMD (g/cm^2^)	0.72	0.12	0.71	0.12	0.312

L-BMD percent changes during late treatment in the TPTD group and Placebo group were 5.8 ± 4.6 % (*n* = 113) and 0.6 ± 3.8 % (*n* = 138) at 48 weeks and 6.7 ± 5.3 % (*n* = 107) and 0.3 ± 4.5 % (*n* = 130) at 72 weeks, respectively, showing significant differences between the groups at both time points (*p* < 0.001).

**Fig. 1:**
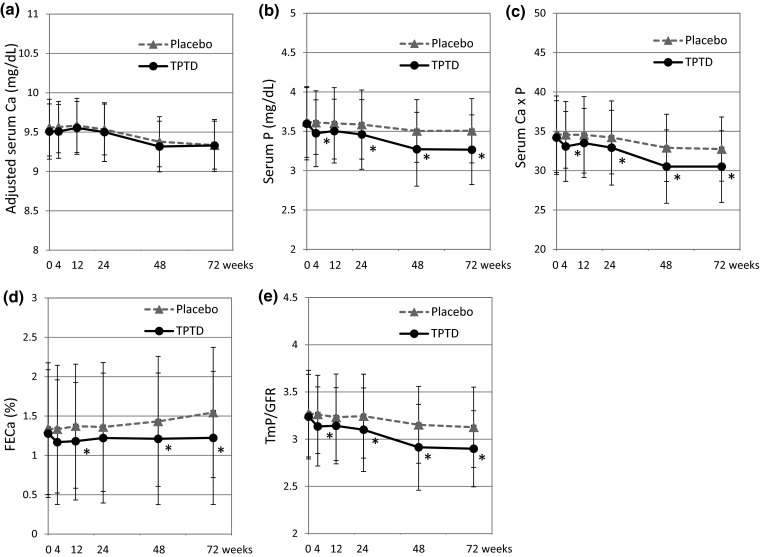
Changes of calcium, phosphate, and the reabsorption rate. **a** adjusted serum calcium, **b** serum phosphate **c** serum calcium × phosphate, **d** urinary fractional calcium excretion (FECa) and **e** phosphate reabsorption index (TmP/GFR). *TPTD* teriparatide. * *p* < 0.05, values are mean ± SD

Adjusted serum calcium, phosphate, calcium-phosphate product, FECa, and TmP/GFR at each time point are shown in Fig. 1. Serum calcium was not significantly different between the groups. Serum phosphate, calcium-phosphate product, and TmP/GFR were significantly lower in the TPTD group at weeks 4, 24, 48, and 72 (*p* < 0.05). FECa was significantly lower in the TPTD group at weeks 12, 48, and 72 (*p* < 0.05).

Table 2 shows the correlation between adjusted serum calcium, phosphate, calcium-phosphate product, FECa, and TmP/GFR in the TPTD group during early treatment (weeks 4 and 12) and the percent change of L-BMD during late treatment (weeks 48 and 72). Serum phosphate and TmP/GFR at both 4 and 12 weeks showed a significant positive correlation with the percent change in L-BMD at both 48 and 72 weeks (*p* < 0.05).

**Table 2:** Correlations of calcium and phosphate with the change in L-BMD

Parameter	Weeks	Change in L-BMD at 48 weeks		Change in L-BMD at 72 weeks	
		R	*p*	R	*p*
Adjusted serum calcium	4	−0.114	0.230	0.026	0.792
	12	−0.134	0.159	−0.059	0.550
Serum phosphate	4	0.215	0.022	0.205	0.034
	12	0.233	0.013	0.218	0.024
Adjusted serum calcium × phosphate	4	0.163	0.084	0.186	0.055
	12	0.162	0.087	0.169	0.082
FECa	4	−0.010	0.920	0.001	0.991
	12	−0.015	0.877	−0.037	0.705
TmP/GFR	4	0.231	0.014	0.228	0.019
	12	0.288	0.002	0.312	0.001

**Fig. 2:**
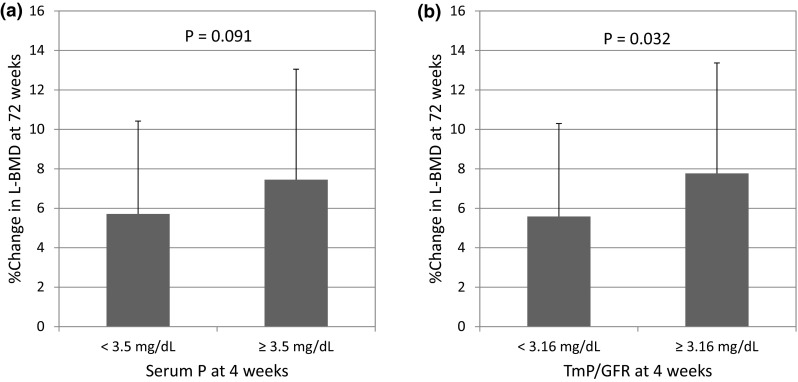
Differences in changes of L-BMD by serum phosphate and TmP/GFR category. **a** serum phosphate and L-BMD and **b** TmP/GFR and L-BMD

Table 3 shows the results of the multivariate analysis using serum phosphate and TmP/GFR, which are variables that showed significant differences during early treatment (week 4). Based on the analysis adjusted for age, BMI, and initial L-BMD, serum phosphate and TmP/GFR at week 4 showed a significant association with the percent change of L-BMD (*p* < 0.05). Serum phosphate and TmP/GFR at week 4 were split at the median (3.5 and 3.16 mg/dL, respectively) into high and low groups, and the percent changes in L-BMD at 72 weeks were compared between these groups (Fig. 2). Greater L-BMD was observed in the serum phosphate ≥3.5 mg/dL group, though the difference was not significant, and significantly greater L-BMD was observed in the TmP/GFR ≥3.16 mg/dL group (*p* = 0.032).

**Table 3:** Multiple regression analysis for change in L-BMD at 72 weeks

Model	Item	Estimate	SE	*p*	R^2^
1	Age	−0.01	0.09	0.890	0.090
	BMI	0.29	0.17	0.091	
	L-BMD at 0 weeks	−0.10	0.05	0.041	
	Serum phosphate at 4 weeks	2.46	1.21	0.044	
2	Age	−0.03	0.09	0.783	0.094
	BMI	0.29	0.17	0.089	
	L-BMD at 0 weeks	−0.09	0.05	0.068	
	TmP/GFR at 4 weeks	2.63	1.21	0.032

## Discussion

In the present study, the changes in calcium, phosphate, calcium excretion, and phosphate reabsorption were examined in patients who received once-weekly long-term TPTD treatment. With TPTD, serum phosphate and calcium-phosphate product decreased significantly. Moreover, the calcium excretion index FECa and the phosphate reabsorption index TmP/GFR also decreased. The associations between these decreases and the percent change in L-BMD were also examined during late TPTD treatment to demonstrate that Tmp/GFR was positively correlated with the percent increase in L-BMD. These results indicate that the smaller the decrease in renal tubule phosphate reabsorption with once-weekly TPTD treatment, the better it is for increasing BMD. These were the first observations indicating the clinical significance of adequate phosphate response to TPTD administration for more efficient acquisition of BMD during once-weekly TPTD treatment. In addition, they suggest the clinical importance of monitoring serum phosphate levels during TPTD therapy.

When TPTD is given, there is a transient elevation in the serum calcium concentration and a transient decrease in the phosphate concentration, both of which return to their respective baseline levels by the subsequent TPTD treatment [13]. However, the present results suggest that the decrease in renal phosphate reabsorption initiated by TPTD administration lasts for a long time in quite a few of subjects. Given that the biological actions of exogenous PTH after a single TPTD injection do not continue over a day because circulating levels of TPTD returned to basal value until 7 h after injection [13, 14], the decrease in serum phosphate is possibly due to the decrease in Tmp/GFR that might be caused by some intrinsic mechanisms, such as endogenous PTH, FGF23, and vitamin D actions that regulate phosphate metabolism. For example, exogenously administered TPTD has been shown to increase the circulating level of FGF23 [16], which could inhibit renal phosphate reabsorption and vitamin D activation. Thus, once-weekly TPTD administration might decrease serum phosphate levels via the stimulation of FGF23 when vitamin D actions are insufficient to prevent its decrease, for instance, in the presence of vitamin D insufficiency/deficiency. Although mean values of serum endogenous PTH were suppressed by TPTD administration until 6 days [13], endogenous PTH levels might also be dependent on vitamin D sufficiency. Then, another possibility is that relatively lower serum phosphate levels might be observed in subjects with relatively higher endogenous PTH levels in the presence of vitamin D insufficiency/deficiency.

With regards to TPTD’s effects on bone, it is known that bone formation is promoted via TPTD’s effects on the stimulation of osteoblast progenitor proliferation [17]. Another effect of TPTD on bone is the promotion of bone resorption mediated through RANKL expression stimulation [18]. The primary effects of TPTD from the perspective of calcium and phosphate metabolism are the promotion of bone resorption and renal calcium reabsorption and the suppression of renal phosphate reabsorption. TPTD, therefore, could have dual effects, regulation of calcium and phosphate metabolism via its physiological actions on bone and kidney and bone formation via its pharmacological actions on bone-forming cells. Therefore, when primarily considering bone formation, because appropriate levels of calcium and phosphate are necessary for the mineralization of the bone matrix, the decrease in serum phosphate concentration is postulated to be unfavorable in bone mineralization, resulting in less BMD acquisition. Indeed, osteomalacia caused by decreased bone mineralization is sometimes observed in patients with severe primary hyperparathyroidism or in patients with secondary hyperparathyroidism undergoing dialysis [19].

Since renal tubule phosphate reabsorption decreased with TPTD administration in the present study, a significant decrease in serum phosphate concentration was observed. The degree of this decrease might affect the extent of bone matrix mineralization reflecting BMD, because there was a positive correlation between Tmp/GFR and the percent increase in L-BMD with TPTD treatment. This suggests that the greater the decrease of phosphate reabsorption with TPTD treatment, the less beneficial it is to BMD elevation. It is unlikely that the direct effects of TPTD given once a week persist until immediately prior to the subsequent treatment. Therefore, it is postulated that the greater the re-elevation of endogenous PTH after its transient suppression with TPTD treatment, the lower the phosphate reabsorption immediately before TPTD treatment and the less the increase in BMD. Otherwise, once-weekly TPTD could repeatedly stimulate the secretion of FGF23 that inhibits renal phosphate reabsorption and vitamin D activation and might decrease serum phosphate levels via the stimulation of FGF23, when vitamin D stores are insufficient due to vitamin D insufficiency/deficiency.

In general, the factor that is considered to most strongly affect circulating PTH levels in individuals with normal renal function without abnormal parathyroid function is vitamin D status [20, 21]. This signifies that, since circulating PTH increases in response to vitamin D deficiency or insufficiency, sufficient vitamin D levels appear to be necessary to maintain PTH at a physiologically stable state.

Our findings suggest two distinct biological functions of TPTD and PTH, bone formation promotion and calcium-phosphate metabolism regulation, and that both of them act independently from one another. In addition, it is possible from the perspective of BMD elevation that a sufficient vitamin D level is necessary to suppress the promotion of endogenous PTH secretion and to minimize the decrease in serum phosphate concentration during TPTD treatment. The present results suggest that further increases in BMD may be attained by more vitamin D supplementation when there is a marked decrease in serum phosphate concentration during TPTD treatment. Alternatively, larger and/or longer suppression of endogenous PTH by TPTD might be involved in greater BMD acquisition along with less decrease in serum phosphate irrespective of vitamin D sufficiency.

There are several limitations to this study. First, this study did not measure the endogenous concentration of PTH. It is therefore not clear whether the present results are a direct effect of TPTD or an indirect effect mediated via endogenous PTH. Second, 25-hydroxy vitamin D concentration before TPTD treatment was not measured, and the state of vitamin D sufficiency was, therefore, unknown. Moreover, while patients received native vitamin D supplementation, the amount was relatively low at 400 IU/day, and whether this amount adequately improved vitamin D deficiency or insufficiency is not known.

In summary, this study suggests that the L-BMD response to once-weekly long-term TPTD treatment is associated with circulating phosphate and the status of its renal reabsorption. To prevent the decrease in the serum phosphate level, for example, with ample vitamin D supplementation, may be important in acquiring greater BMD with once-weekly TPTD.
